# Comparing Two Surgical Approaches Using Cross-Linked Hyaluronic Acid-Biofunctionalized Alloplast Particulate in Sinus Floor Elevation: A Randomized Clinical Trial

**DOI:** 10.3390/jfb17020086

**Published:** 2026-02-09

**Authors:** Chantal Wittmers, Anton Friedmann, Andreas van Orten, Bashar Husseini, Werner Götz

**Affiliations:** 1 Department of Periodontology, Faculty of Health, School of Dentistry, Witten/Herdecke University, Alfred-Herrhausen-Str. 50, 58455 Witten, Germany; chantalszcz@gmail.com; 2 Private Office, 45731 Waltrop, Germany; 3 Dental Square Clinic, Beirut, Lebanon; 4 Department for Orthodontics, Friedrich-Wilhelm-University Bonn, 53113 Bonn, Germany; wegoetz28@gmail.com

**Keywords:** bone grafting, cross-linked hyaluronic acid, ß-TCP/HA, dental implants, sinus floor elevation

## Abstract

**Objective:** The purpose of this study was to assess the outcome of sinus grafting with a beta-tricalcium phosphate/hydroxyapatite (ß-TCP/HA) alloplast particulate biofunctionalized with cross-linked hyaluronic acid (xHya), comparing two surgical access techniques. Clinical, histological, histochemical, immunohistochemical and histomorphometrical parameters were used to characterize the tissue samples, which were retrieved at the second surgery for implant placement five months after sinus floor elevation (SFE). **Materials and Methods:** Twenty patients with a residual bone height ≤ 4 mm, estimated by a Cone Beam Computed Tomography (CBCT), were randomly allocated either to an innovative transcrestal sinus floor elevation (tSFE = tests) approach or a conventional lateral window approach (lSFE = controls) using piezoelectric preparation. The tSFE was carried out using the hydraulic Jeder^®^-System. Grafting in both groups was performed using a ß-TCP–HA combination, which was biofunctionalized with a cross-linked hyaluronic acid. For both access techniques, a cross-linked collagen membrane covered either the bone window or transcrestal osteotomy. For second-stage surgery, a second CBCT was used to assess the bone volume and possible implant positioning to compare it with the baseline CBCT. Bone cores were harvested at implant placement and evaluated histomorphometrically. Patients were followed for 1-year post-op for survival rate estimation. Non-superiority was hypothesized for both surgical methods; thus, the primary outcome measure assessed different discomfort levels using patient-reported outcome measures (PROMs) for each therapeutic approach. Secondary outcomes were the volume change in subantral bone after sinus floor elevation, the chance of placing a 10 mm long implant with no need for additional augmentation, histological evaluation of the newly gained tissue, and implant integration and one-year survival. **Results:** Eighteen patients (*n* = 18/20) qualified for implant placement at five months, and ten donated tissue biopsies for microscopic analysis. Primary outcome reporting using PROMs was discarded due to truncated patient enrollment. The secondary parameter, placement of a ≥10 mm long implant without additional augmentation, was achieved for nine sites/patients from the lSFE control group. All patients from the tSFE test group received an implant that was positioned alongside additional augmentation. In both groups, all implants integrated and were functionally loaded. A total of 10 core samples (3 from the tSFE group and 7 from the lSFE group) were obtained and analyzed. Microscopically, new bone formation appeared consistent in all obtained samples. Specimens revealed advanced and ongoing osteogenesis, with most histological markers reacting positively in the immunohistochemical (IHC) staining. The histomorphometric calculation revealed that a mean of 61.17 ± 16.55% of the total area was occupied by newly formed bone, 30.43 ± 10.09% by connective tissue and 8.92 ± 15.29% by residual graft substitute. One-year follow-up of the loaded implants showed a 100% implant survival rate. **Conclusions:** Biofunctionalizing ß-TCP + HA particulate with cross-linked hyaluronic acid in sinus floor elevation procedures appears to be a safe and beneficial approach, resulting in satisfactory clinical, radiographic and histological parameters. In our study population, which presented with very atrophic residual subantral bone conditions, the hydrodynamic transcrestal sinus floor elevation method required a back-up treatment by the conventional lateral approach.

## 1. Introduction

Maxillary sinus floor elevation (SFE) is a surgical technique that aims to compensate for vertical bone loss after a dental extraction or unassisted tooth loss in order to place a dental implant.

In the 1970s, Hilt Tatum introduced an external technique for gaining direct access to the maxillary sinus through the lateral osteotomy window, which Boyne and James first published in 1980. The authors suggested raising the Schneiderian membrane and placing a bone marrow filling under the elevated membrane [1]. Summers et al. introduced a technique for a transcrestal approach in 1994, suggesting the use of concave tipped osteotomes to break the sinus floor and elevate the Schneiderian membrane, with a minor risk of membrane perforation [2]. Several clinical trials showed that, for both techniques, accurate prediction of a safe outcome relates to the residual subantral bone height [3,4,5,6,7]. Meanwhile, any kind of SFE competes with the placing of a short or ultrashort implant [3,5]. However, once a new technique for a crestal approach is proposed, the reference for its performance remains the lateral window approach [7,8,9,10].

However, both surgical methods for uplifting the sinus membrane require its suborbital stabilization to maintain room for bone ingrowth [11,12]. This stabilization may be obtained by the implants themselves protruding into the sinus cavity as posts and supporting the membrane by their apical tip [13,14,15,16,17]. However, the most common technique uses bone substitutes and/or autologous bone for grafting the space instead, either simultaneously to implant placement or as a step prior to this. The nature of bone grafts remains a contested topic, while autogenous bone is still considered the gold standard due to its osteogenic properties. Its limited quantity, fast resorption pace and post-operative morbidity maintain its status as favored over other bone substitutes. These bone substitutes—allografts, xenografts and alloplasts—have osteoconductive properties in common [18,19,20]. Alloplasts based on a beta-tricalcium phosphate (β-TCP) structure with a relatively fast resorption profile were introduced, with the suggestion of a similarity between resorption kinetics and the bone formation rate [21]. In theory, low-temperature-sintered β-TCP may develop osteoinductive properties and resorb alongside bone apposition, thus leaving the grafted area filled by newly native bone. The strategy of covering the access window with a membrane is a common procedure, with the aim of disconnecting the grafted area from the soft tissue flap and stabilizing the graft against pneumatic pressure from the Schneiderian membrane [22]. Ribose cross-linked collagen membranes are helpful for this purpose but are also used in an open healing situation to cover the orifice after tooth extraction [23]. Hyaluronic acid, a major component of the extracellular matrix, is considered one of major promotors of wound healing in general and is currently being used as a biological adjunctive in bone augmentation due to its regenerative properties, such as growth factor attraction, accelerated angiogenesis, hydrophilicity and matrix stabilization [24]. Cross-linked hyaluronic acid (xHyA) exhibits a plethora of beneficial properties for the repair and reconstruction of oral tissues in both soft- and hard-tissue injuries [25,26,27,28]. Clinical case series demonstrated that biofunctionalizing diverse surfaces and substrates using xHyA resulted in enhanced healing responses. Thus, an implant with a history of periimplantitis and a tooth suffering from periodontitis both improved impressively at the adjacent Marginal Bone Level (MBL) once they were treated surgically by a combination of xHyA and either a membrane or collagen matrix [29,30]. A randomized controlled trial (RCT) used a split mouth model to compare two methods for the grafting of the sinus using hyaluronic acid: mixing with putty ß-TCP paste (test) and mixing with particulate ß-TCP (control). The histomorphometric results were in favor for the test side in the seven cases reported [31]. We hypothesized that a beta-tricalcium phosphate/hydroxyapatite (β -TCP/HA) bone graft that is biofunctionalized with xHyA may withstand rapid degradation by accelerating the bone apposition.

The aim of this study was to evaluate the effect of biofunctionalizing a particulate alloplast graft with xHyA by clinically, radiographically, histologically and histomorphometrically accessing the sinus cavity following two surgical methods.

## 2. Materials and Methods

### 2.1. Study Design

This study was conducted as a prospective, monocentric, single-blinded and two-armed randomized controlled trial (RCT). The protocol of the study was approved by the Ethics Committee of Witten/Herdecke University (163/2018/2019), and an informed consent form was signed by each patient before enrollment into the study according to the Helsinki declaration and its ethical requirements from 2008. The study ID registered at DRKS is DRKS00039034. The CONSORT checklist is attached as a complementary file. Both therapy concepts were randomized in a 1:1 relation assuming non-superiority between the two methods for accessing the sinus cavity. Mean scores of 22 points for the control group and 29 points for the test group based on the Oral Health Impact Profile-14 (OHIP-14) questionnaire were expected, with a standard deviation of 6 versus 12 points, respectively [32]. To reveal this difference with a minimum power of 80% and an α = 0.05, 31 patients were needed in each group. Considering a 20% drop-out rate in the follow-up period, a total of 37 patients for each group was calculated.

The clinical part of the study was conducted between 2019 and 2021. All patients underwent sinus floor elevation (SFE), followed by five months of healing prior to re-entry surgery and implant placement. Implant survival was assessed one year after functional loading.

After completion of the second-stage surgery for the first 20 enrolled patients, further recruitment was truncated. This decision was based on a consistently insufficient subantral bone gain and pronounced graft resorption in the transcrestal SFE group, which served as the test group. Thus, patient-reported outcomes assessed using PROMs were discarded as the primary endpoint.

Patients who were recruited for the study presented with a maximum subantral alveolar bone height of 4 mm; thus, in both groups, staged sinus grafting was proposed, in agreement with the international guidelines. Table 1 reports the patients’ demographic data in detail. The secondary parameter, the amount of subantral bone that was sufficient for a straightforward implant placement without the need for additional grafting, was the next objective to fulfill. Assessed by radiographic measures, the subantral bone fill that was displayed in the second Cone Beam Computed Tomography (CBCT) after a healing period was contrasted with the bone contours from the baseline CBCT. Secondary outcomes were related to the microscopically assessed composition of the newly formed tissue, using a combination of a synthetic and fully degradable bone substitute and cross-linked hyaluronic acid (xHya).

The random allocation of surgical technique was performed using the sealed envelope model, allocating patients to transcrestal hydrodynamic sinus floor elevation (tSFE) or the lateral window approach (lSFE), respectively. A blinded investigator (C.W.) opened the sealed envelope and informed the operator (A.v.O.) of a patient’s allocation to one of the respective treatments before the scheduled surgery started. During surgery, this investigator was absent, thereby remaining blinded to the procedure that was executed. The investigators (C.W., W.G.) who were responsible for microscopic analysis stayed blinded to the type of surgical approach till the end of the evaluation. Following the inclusion and exclusion criteria shown in Table 1, twenty patients were enrolled in this study. All surgeries were performed by the same operator (A.v.O.) at a private dental clinic.

### 2.2. Surgical Technique

Before each surgery, a CBCT was taken for each patient to assess their sinus anatomy, as well as to document a precise baseline subantral bone extension for later radiographic evaluation.

As a pre-medication, either 2 g of amoxicillin or 600 milligrams of clindamycin was administered one hour before the surgery. All surgeries were carried out under local anesthesia (Ultracain^®^ D-S forte, Sanofi-Aventis Deutschland GmbH, Frankfurt, Germany), and patients were placed under sedation with Midazolam (Ratiopharm^®^ GmbH, Ulm, Germany) on individual demand.

The incision design was performed depending on the access technique. Thus, an intrasulcular mesio-distal incision in two teeth alongside two vertical incisions were performed in patients who were allocated to the lateral window SFE group, while a simple midcrestal gingival punch was sufficient for the transcrestal approach.

After flap elevation using an ultrasonic device (Piezosurgery^®^, Mectron Deutschland Vertriebs GmbH, Köln, Germany), we created lateral access, and the Schneiderian membrane was elevated using sinus elevation curettes. In the crestal approach, the membrane was elevated by hydraulic pressure after a pressure chamber (Jeder^®^-System, Jeder GmbH, Vienna, Austria) was attached, once the gingiva punch was removed [33]. The device was then sealed sufficiently to create a minimum of 1.2 bar water pressure (isotonic 0.9% NaCl- solution, B. Braun Melsungen AG, Melsungen, Germany) at the residual subantral bone to initiate the infraction of the sinus floor. After elevating the Schneiderian membrane and evacuating the saline solution from the sinus cavity, the osteotomy at the crestal bone level was continued by a round piezoelectric insert. In case of membrane rupture, the perforation was closed using either a collagen fleece (Parasorb^®^, Resorba Medical GmbH, Nürnberg, Germany) or periacryl biologic adhesive (Periacryl 90Hv, American Dental Systems GmbH, Vaterstetten, Germany), and the patient was excluded from the study.

The grafting of the sinus cavity was carried out by applying an alloplastic particulate composed of low-temperature-sintered beta-tricalcium phosphate/hydroxyapatite (β-TCP/HA) at a ratio of >90%/<10% and with a particle size of 0.25–1.0 mm (Osopia, Regedent GmbH, Dettelbach, Germany), which had been re-hydrated by a normalized amount of cross-linked hyaluronic acid gel (xHyA; Hyadent BG, Regedent GmbH, Dettelbach, Germany). This sticky bone mixture was gently condensed into the sinus cavity in both groups. According to the protocol, the tSFE group received 2.0 cc of the particulate per site, whereas the amount required for the lSFE group was not standardized. The total mean for the graft material inserted was therefore 2.72 +/− 1.07 cc. In each case, the antrostomy was covered with a resorbable porcine sugar cross-linked collagen membrane (OSSIX^®^ Plus, Regedent GmbH, Dettelbach, Germany). The flap was then repositioned and sutured using a 5.0 non-resorbable monofilament (Propylen 5.0, Medipac^®^ GmbH, Königswinter, Germany). The suture removal was scheduled after 7 days in both groups. The tSFE group received a ribose cross-linked collagen membrane covering the soft tissue punch area for graft stabilization until implant installation, which was performed for all participants 5 months later.

For post-operative medication, amoxicillin 1000 mg or clindamycin 300 mg was prescribed three times daily for 7 days, non-steroidal anti-inflammatory drugs (Ibuprofen 800 mg) were recommended depending on individual needs, and chlorhexidine mouthwash two times daily was recommended for 7 days.

The CBCT was repeated after 5 months using the same CBCT device (Orthophos^®^ XG, Dentsply Sirona, NC, USA) as for the first one to determine the amount of vertical and volumetric ridge alteration. Regardless of whether the re-entry alongside implant placement took place, the scan was performed to estimate the amount of newly mineralized tissue that had formed. If appropriate, tissue biopsies were retrieved from the crest of the ridge using a trephine bur. An experienced operator (A.v.O.) performed the second-stage surgeries at a private dental clinic, without restrictions on the choice of implant system. Twenty-three dental implants with a minimum length of 10 mm (18 ICX Active Master implants, Medentis medical GmbH, Germany) and five Conelog^®^ implants (Camlog Vertriebs GmbH, Wimsheim, Germany) were inserted, and two months later, zirconia cementum-retained crowns were delivered.

### 2.3. Histological Preparation

Following sample harvesting, the core biopsies were fixed in 4.5% phosphate-buffered formaldehyde. Biopsies were decalcified for 12 weeks in 10% Ethylenediaminetetraacetic acid (EDTA) and then dehydrated in graded alcohol baths (70–100%) and xylol (Merck, Darmstadt, Germany). After becoming embedded in plastic cuvettes by a paraffin-pouring station (Sakura, Heppenheim, Germany), the biopsies were cut into 3.5 μm sections using a rotation microtome, HM 355 S Micro International (Microm International GmbH, Walldorf, Germany), and positioned on separate object carriers (D Superfrost Plus, Menzel- Gläser, Braunschweig, Germany). To prepare for the staining, the sections were deparaffinized with the xylol substitute XEM 2000 (Vogel, Gießen, Germany), graded alcohol (100–70%) and distilled water.

For a global preview of the grafted area, Hematoxylin–Eosin (H.E.) staining was applied on several sections. For histochemistry, staining for Periodic Acid–Schiff (PAS) and tartrate-resistant acid phosphatase (TRAP) was performed. Immunohistochemistry (IHC) was carried out by using antibodies against alkaline phosphatase (ALP), collagen type 1 (COL1), ED-1, Osteocalcin (OC), Osteopntin (OP), runt-related transcription factor 2 (RUNX2) and von Willebrand factor (vWF). Antibody binding was visualized using the EnVision method. An experienced examinator (W.G.) analyzed the sections qualitatively at the University Hospital in Bonn using a light microscope (Axioskop 2, Zeiss, Jena, Germany). Representative regions of interest (ROIs) were defined for semi-quantitative evaluation and assessed at 5-, 10-, 20- and 40-fold magnification. Photographic documentation was performed using a microscope camera, AxioCam MRc, and the AxioVision Rel. 4.5” software from Zeiss. ROIs were selected after histological evaluation for the following regions: perigranular osteogenesis, vital bone with remodeling zones and connective tissue. The specimens were then transferred to the University of Witten/Herdecke in Germany and digitalized by a microscopy slide scanner Fritz (PreciPoint GmbH, Freising, Germany) using a 20-fold objective and a resolution of 10,000 pixels for histomorphometrical analysis.

### 2.4. Histomorphometric Assessment Technique

H.E. slices were imported to a specialized software (Image-J, NIH, USA) to perform semi-automatic quantification. First, images were adjusted according to a given scale, and then the background was eliminated, allowing for a proper surface measurement of the total surface. Newly formed bone (NFB) was distinguished from connective tissue (CT) and residual graft (RG) particles, using a threshold filter. For better accuracy, measurements were performed twice: once using the RGB (red, green, blue) filter and once using the HSB (hue, saturation, brightness) filter. Following the surface identification process ascribing findings to either NFB or CT, the area occupied by RG particles was calculated using the following equation: S_residual particles_ = 100 − (S_newly formed bone_ + S_connective tissue_).

### 2.5. Statistical Analysis

For the statistical analysis, Word, Excel (Microsoft, Seattle, WA, USA) and R Studio (Version 2021.09.2; RStudio PBC, Boston, MA, USA) were used. Most data were presented as mean ± standard deviation (SD) and median. The categorical variables were described using count and percentage. Only the molar areas were used for statistical analysis. Due to the limited study population, only descriptive analysis was applied.

## 3. Results

### 3.1. Study Population

**Figure 1:**
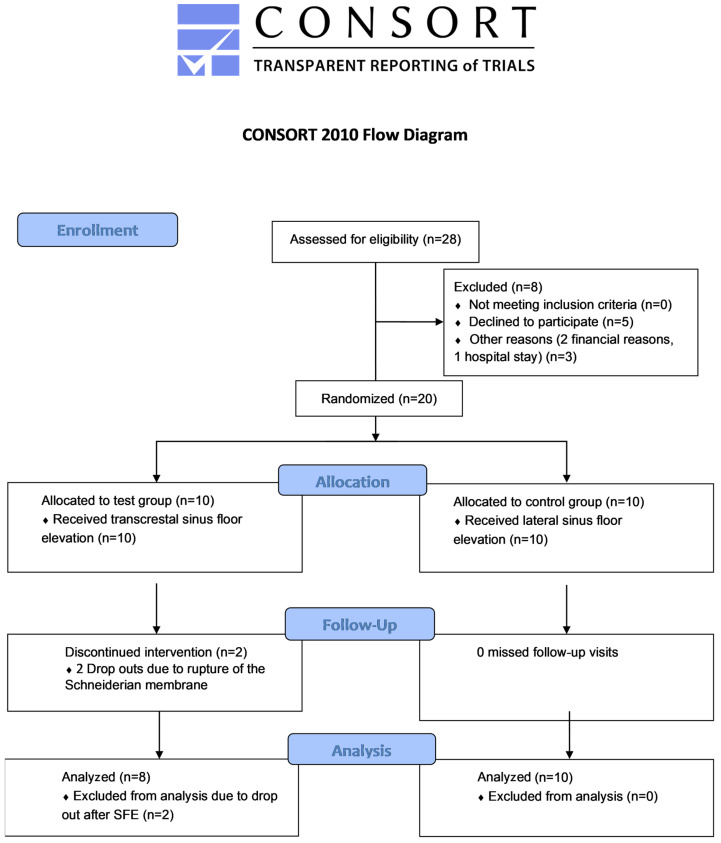
Flowchart of the RCT.

The study population consisted of 20 patients, who were randomized into two groups. However, two patients from the tSFE (test) group experienced a rupture of the Schneiderian membrane during the procedure and were classified as dropouts for violating the protocol (Figure 1, CONSORT flow chart). Two patients from the lSFE (control) group also experienced a membrane rupture; however, according to the protocol, these were not considered violations (Table 2).

Thus, 7 females (39%) and 11 males (61%) with a mean age of 58.39 ± 9.21 years were followed in the post-op period. The mean residual bone height (RBH) at baseline was 2.5 ± 0.88 mm. The mean ridge height score was 11.0 ± 5.44 mm, with an average gain of 8.5 ± 5.57 mm according to our comparison of the baseline and 5-month CBCT (see Table 3). All sites from the tSFE group had to be re-augmented at the second surgery, as implants were inserted. All sites in the tSFE group failed to reach our primary outcome.

The number of available core biopsies was 10, 7 of which resulted from lSFE and 3 from the tSFE procedure. As revealed by qualitative observations, the specimen composition demonstrated significant similarity between samples from both access techniques. Thus, specimens were pooled to calculate the histomorphometric quantification numbers in a total population. The ten donors received an implant placement concomitantly to core biopsy retrieval, without additional bone augmentation. The eight remaining patients presented with limited vertical bone dimension at re-entry; thus, their participation in the further analysis was discarded.

These eight patients underwent additional sinus grafting procedures and were listed as failures in terms of the primary outcome. The enrollment of further patients for random allocation within two groups was truncated, as the dominant complication rate for the tSFE group became evident.

**Figure 2:**
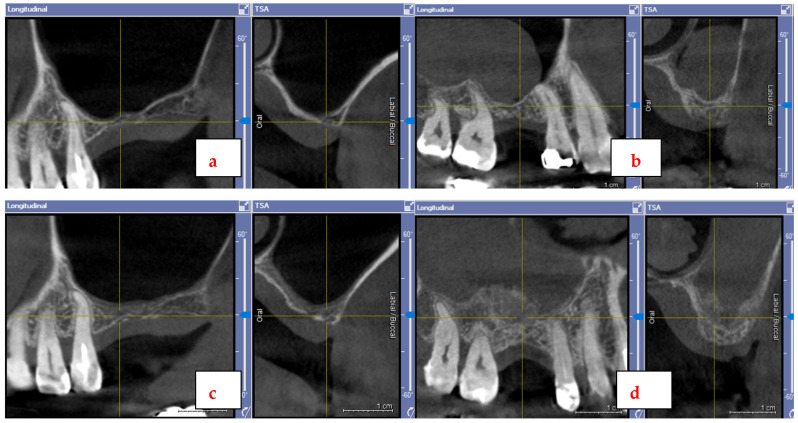
CBCT scans of a test group (**a**,**c**) and control group (**b**,**d**) participant before and after SFE.

Figure 2 demonstrates a representative pair of CBCTs from baseline and 5 months after SFE, indicating that almost no gain in additional bone occurred in the test site (Figure 2a–c), while the control site presents with an impressively improved bone volume (Figure 2b–d). The second cone beam assessment consistently documented a rapid loss of grafted area in the test group but a severe gain in volume for controls.

### 3.2. Histological Observation and Histomorphometry Analysis

**Figure 3:**
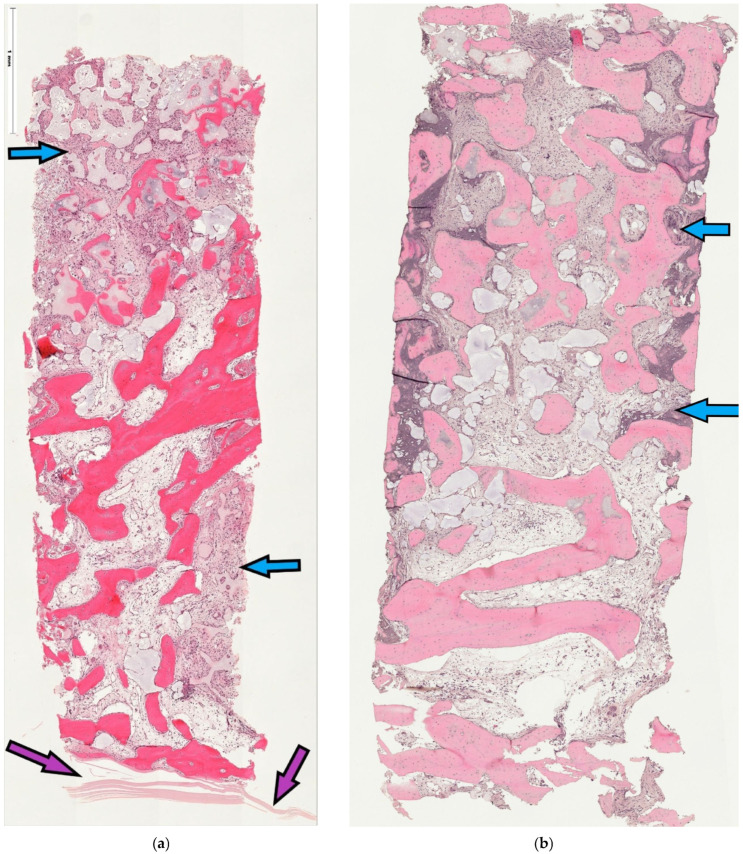
(**a**,**b**) Complete core cross-sections from tSFE (**a**) and lSFE sinus grafting (**b**) (H.E. stain). (**a**) H.E. stain of representative core biopsy from tSFE group. Purple arrows point to residues of cross-linked collagen membrane which covered the graft inserted transcrestally, while blue arrows indicate the graft area extension; original magnification ×5. (**b**) H.E. stain of a representative core biopsy from lSFE group. Blue arrows indicate extension of grafted area; original magnification ×5.

Microscopic analysis revealed consistent formation of new bone in all available samples. Furthermore, specimens appeared consistently well vascularized, with areas of both woven and lamellar bone being present, as well as displaying various degrees of appositional bone growth to the particle graft residues. Residual graft (RG) was found in almost every biopsy in varying quantities, and most RG particles appeared to be embedded in newly formed bone (NFB). Areas showing bone remodeling were ubiquitous. The pattern of graft and new tissue allocation within the specimen varied in relation to the access technique for approaching the sinus cavity. Thus, specimens retrieved from samples after tSFE demonstrated remnants of cross-linked collagen membrane with a thin layer of new trabecular bone attached to and located underneath it, followed by an area that was occupied by the graft residues and newly formed tissue (Figure 3a). Following the lSFE procedure, the area that was occupied by graft and newly formed tissue was located more cranially, underneath a layer of residual subantral native bone (Figure 3b). Histomorphometric analysis revealed a mean of 61.17 ± 16.55% ROIs being occupied by NFB, while 30.43 ± 10.09% were occupied by connective tissue (CT), and RG occupied 8.92 ± 15.29% of the area in all 10 specimens (Table 4). The grade of impaction of graft residues into new bone appeared greater in the lSFE group; however, the histomorphometric outcome reported below failed to identify superiority for either group.

**Figure 4:**
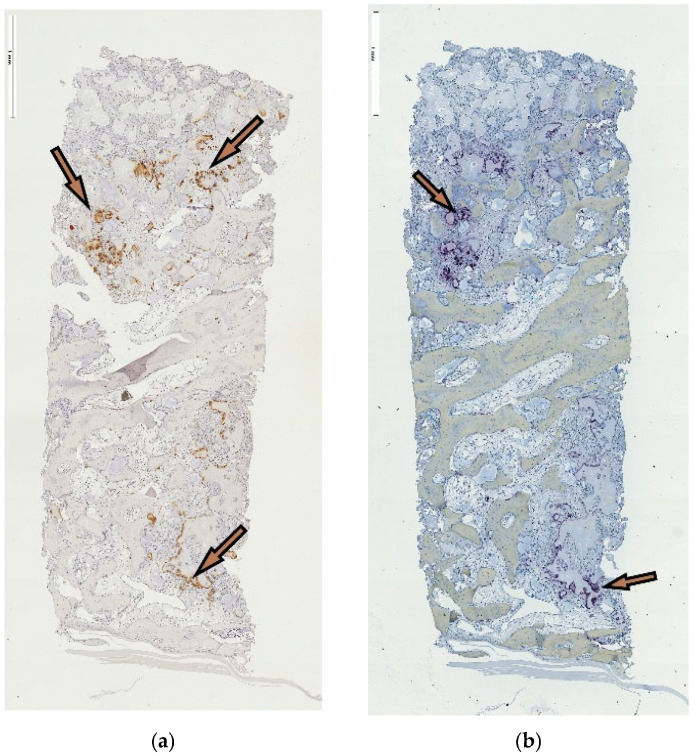
(**a**,**b**): ED-1 and TRAP reaction in tSFE group. (**a**,**b**) ED-1 and TRAP reactions in the tSFE group indicate co-localization of positively reacting cells in IHC, identifying the differentiated active osteoclasts, original magnification ×5.

**Figure 5:**
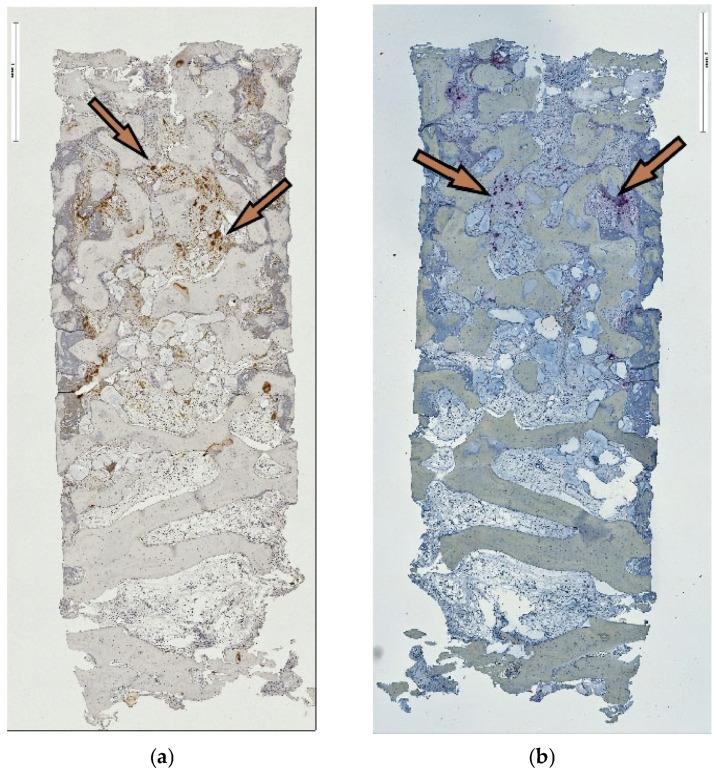
(**a**,**b**) ED-1 and TRAP reaction in lSFE group. (**a**,**b**) ED-1 and TRAP reactions in lSFE group indicate co-localization of positively reacting cells in IHC, identifying the differentiated active osteoclasts, original magnification ×5.

The spatial similarity localizing the ED-1-labeled cell activity and TRAP-positive reaction at residual graft particles was consistently observed in both the tSFE and lSFE group. However, the distribution of the positively reacting cells in respective areas of the specimens was in agreement with the abovementioned pattern in the area constellation according to the access procedure (Figure 4a,b and Figure 5a,b, respectively). Multinuclear giant cells (MNGCs) were absent. Inflammation extension appeared limited to either a moderate or low level. Mostly loose connective tissue, blood vessels and adipose tissue filled the inter-trabecular marrow.

**Figure 6:**
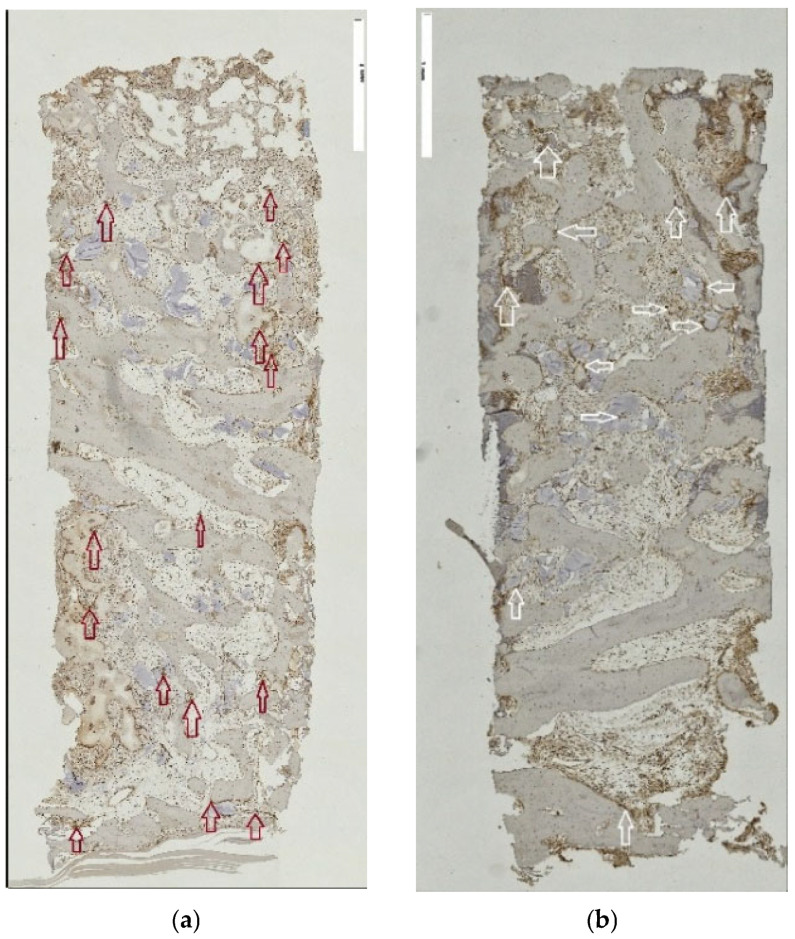
(**a**,**b**) RUNX2 in tSFE and lSFE group. (**a**,**b**) The correlation of positive RUNX2 reactions and the grafted area extension according the grafted access technique shows differentiated osteoblasts ((**a**): tSFE, red arrows; (**b**): lSFE, white arrows), original magnification ×5.

**Figure 7:**
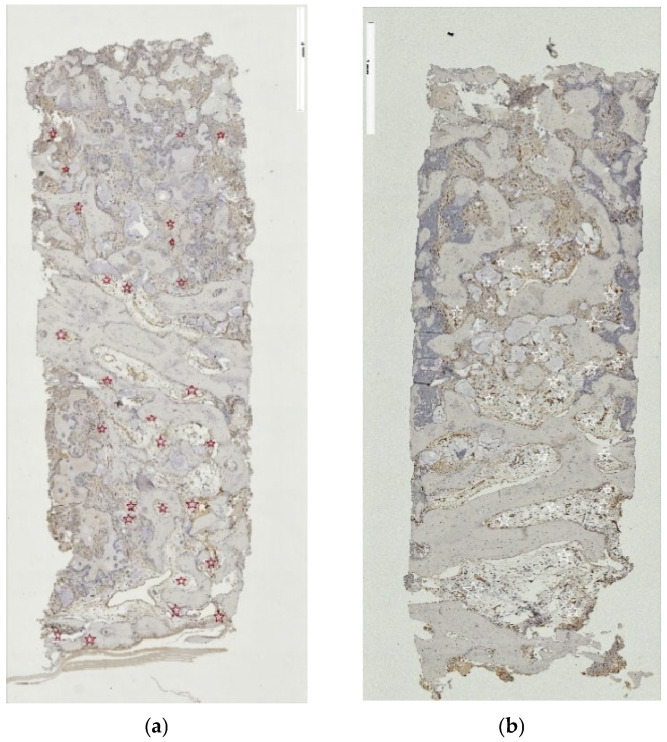
(**a,b**) vWF in tSFE and lSFE group. (**a**,**b**) The similar patterns of vWF reactivity in tissues formed in the grafted areas verifies the vessel presence ((**a**): tSFE, red asterisk; (**b**): lSFE, white asterisk), original magnification ×5.

**Figure 8:**
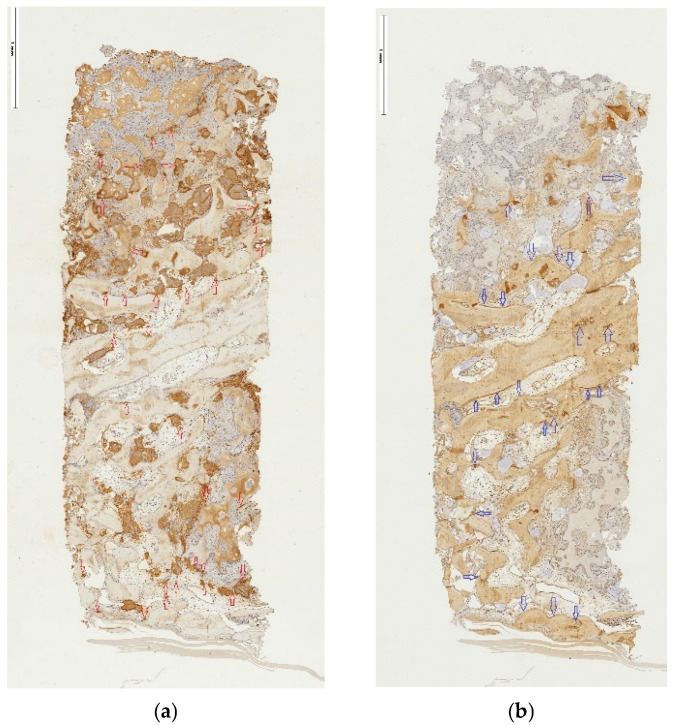
(**a**,**b**) Osteopontin and Osteocalcin in tSFE. (**a**,**b**) Distribution of cells reacting positively with either Osteopontin (**a**) or Osteocalcin (**b**) antibody ((**a**): red; (**b**): blue hollow arrows) in tSFE group, original magnification ×5.

**Figure 9:**
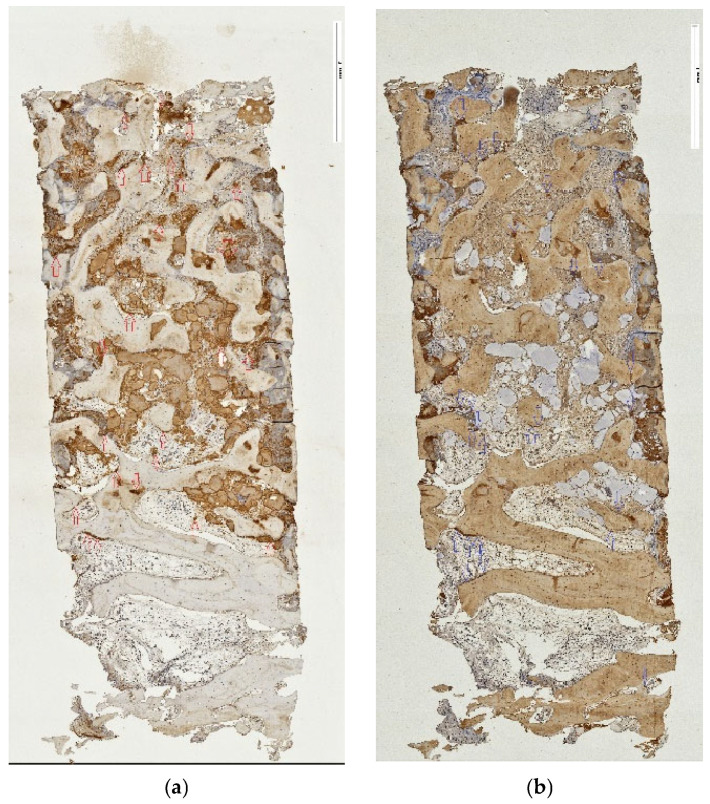
(**a**,**b**) Osteopontin and Osteocalcin in lSFE. (**a**,**b**) Distribution of cells reacting positively either with Osteopontin (**a**) or Osteocalcin (**b**) antibody ((**a**): red; (**b**): blue hollow arrows) in lSFE group, original magnification ×5.

The IHC activity of all bone-encoding proteins, with RUNX2, COL1 and ALP being expressed across almost the total area in every specimen. While the intensity of expression varied from sample to sample for these early bone markers (Figure 6a,b for RUNX2), antibodies against vWF factor consistently documented vascularization in the newly formed tissue, regardless of the access technique (Figure 7a,b). Proteins like OP and OC were present ubiquitously within connective tissue and in the bone matrix, respectively (Figure 8a,b and Figure 9a,b, respectively), and, to a considerably lesser degree, within the bone graft substitute. In addition, OC expression was detected in the osteoblasts. Table 5 summarizes the semi-quantitated observations done on behalf of the IHC reaction for targeting the expression of bone-related proteins and vWF, as well as TRAP-positive cells.

## 4. Discussion

This clinical trial investigated the effect of biofunctionalizing β-TCP/HA with xHyA for SFE. Initially, the study aimed to assess the perception of two surgical techniques using the OHIP-14 questionnaire (PROMs). However, this primary outcome was discarded, as the first ten patients from the test group showed dramatic volume reduction in the previously augmented area and were urgently requiring additional grafting to safely position an implant. From a clinical point of view, implant placement was possible after 5 months in both groups; however, the tSFE patients underwent a secondary SFE procedure parallel to implant insertion. Nevertheless, no implant loss was recorded at the 1-year follow-up. Achieving this outcome in patients with a residual subantral bone height ≤ 4 mm is encouraging, as such cases are often challenging. The conventional lateral window approach worked clinically in accordance with the established outcome rates for this technique. Besides the completely failed primary outcome, two patients in the tSFE group experienced a rupture of the Schneiderian membrane and were both excluded from the study. Therefore, once the first ten patients from the tSFE group completed their second surgery, the study was truncated. However, studies reporting subantral bone volume changes based on either CBCT or panoramic X-ray assessment rarely reveal differences between the lSFE and tSFE approach [8,10,34].

The 10 obtained core samples showed microscopically consistent new bone formation in all biopsy samples, with an average of two thirds of the augmented area being vital bone, regardless of the approach that was chosen for accessing the sinus cavity. The unequal distribution of 3:7 samples from the tSFE vs. lSFE group resulted from the insufficient bone volume in the first group and from some patients declining to donate their tissue in the latter. The residual graft material appeared inconsistently in the specimens, an observation which highlighted that several samples did not contain residues of the alloplast. This outcome underscores that the tested combination of bone substitute and biofunctional modulation provides a favorable remodeling profile within a limited healing period and suggests that the biological performance of the graft material outweighed the difference in access technique. The use of a cross-linked collagen membrane, which is known to resorb slowly, even under open healing conditions where it remains exposed to the oral cavity, may have additionally promoted bone formation in both treatment groups. The residues of this membrane material that were observed in a sample retrieved after the transcrestal procedure (shown in Figure 3a) underscore the longevity and excellent bone-forming capacity of this material. The constantly expressed RUNX2 labeling of active osteoblasts in the specimens suggests that mineralization was ongoing until tissue retrieval, regardless of the surgical access technique. This observation agrees with findings obtained from grafting an extraction site by a collagen cone, which is known to resorb rapidly [35]. The positive and strong vWF expression supported the expectation that the remodeling process was still ongoing after five months of healing in both groups [36].

A plethora of bone substitutes qualified for grafting a sinus cavity when looking at both the clinical and microscopical outcomes. Autogenous bone, allografts, xenografts and alloplasts are known to sufficiently support bone formation within a sinus and are recommended in the literature [19,37,38,39,40,41,42]. The 4 mm RBH, which was thought to represent a threshold for grafting an allograft sinus, did not significantly influence the biopsy assembly that was observed 6 months after using the lateral window approach [43]. Interestingly, in the transcrestal approach, the width of the sinus cavity appeared instead to be related to the numbers reported from histomorphometric assessment of the later tissue composition [44,45]. Apart from these findings, alloplastic proved to be a safe and sufficient biomaterial for substitutions in human bone regeneration [31,46,47,48,49].

The resorption dynamics and duration remain crucial factors for gaining a predictable volume and a sufficient amount of mineralized tissue [50,51]. Thus, the use of non-resorbable biphasic calcium sulfate was associated with almost 41% of the graft particulate being NFB, while the vertical ridge extension was adequately enlarged for placing 10 or more mm long implants after a 6-month healing period [52]. In contrast, the use of quickly degradable alloplastic (Cerasorb) revealed a limited amount of NFB alongside an incomplete remodeling of the graft material, which was regularly observed in the apical half of the specimen [21]. A comparison between the grafting of a sinus cavity with either autologous bone (AB), ß-TCP alone or AB plus ß-TCP in a 1:1 ratio revealed that a significantly greater amount of new bone was formed in the ß-TCP-only grafted group. The IHC staining resulted in similar scorings for RUNX2 and vascular endothelial growth factor (VEGF) expression levels in the ß-TCP-alone and AB grafted groups, while the mixed grafted group presented the highest expression levels of both proteins, although this occurred alongside a significantly smaller amount of NFB [53].

Studies using deproteinized bovine bone mineral (DBBM) have frequently reported residual graft contents between 20 and 30% after 6–9 months, and new bone percentages rarely exceed 40% [39,54,55]. The markedly higher proportion of vital bone in the present paper, together with the low presence of residual particles, suggests that the addition of xHyA to β-TCP may accelerate the turnover of graft material. Earlier investigations have already demonstrated that the addition of hyaluronic acid to xenografts improves their regenerative performance in ridge preservation and lateral augmentation procedures. Besides histomorphometric reports of sinus grafting, our group showed the beneficial effect of xHyA biofunctionalization of bovine xenograft when used either for socket preservation or lateral augmentation in a classic guided bone regeneration (GBR) approach [56,57]. Looking microscopically at the outcome in both the test subjects with xHyA and controls without, these two clinical studies consistently reported a significantly greater area of new bone and a smaller amount of bovine particulate residue in the test subjects. The corresponding porcine particulate graft (DPBM) that had been biofunctionalized with xHyA was shown to sufficiently support bone formation after concomitant grafting of the sinus and the supracrestal bony defect [26]. Furthermore, a histomorphometric comparison of porcine vs. bovine xenografts used for grafting a sinus cavity underscored a beneficial effect of HA pre-treatment of the respective graft [31]. The current findings extend this observation by combining xHyA with the alloplastic substitutes and reinforce the concept that xHyA has a positive biological effect on bone regeneration.

The underlying mechanism may be related to the biological properties of hyaluronic acid itself. As a natural component of the extracellular matrix, it enhances angiogenesis, facilitates the migration of mesenchymal stem cells, and promotes osteoblast differentiation. Cross-linking extends its activity at the grafted site, offering a longer biological window of action. A recent in vitro study highlighted the potential of xHyA to enhance the expression of ALP in osteoblast-like cells in an organoid model. The authors pointed out that the role of xHyA pre-treatment of the collagen material that was used as a substrate for this air-lift model resulted in significantly upregulated ALP contents in the extracellular matrix compared with controls placed on the same substrates without xHyA pre-conditioning [58]. One may speculate that intense vascularization, numerous active osteoblasts and the expression of OP and OC that were consistently observed in our samples suggested a still-progressing bone formation process at the time when the biopsies were retrieved. This assumption may explain the complete substitution of graft material by vital bone within 5 months that was exhibited by some specimens. The upregulation of osteogenic markers that was observed in this study agrees with similar immunohistochemical findings relating to the osteogenesis of other allogenous or synthetic bone substitute materials after SFE [59,60].

This study has multiple limitations: The limited number of patients and biopsies restricts the generalizability of our results. The use of two different approaches may have introduced variability, although the microscopical outcomes were similar in a few samples obtained from both groups. The absence of a control group who were grafted with the same alloplast but without xHyA biofunctionalization restricts the microscopical findings. Moreover, the follow-up of implants placed in this study was limited to 1 year, which does not allow for conclusions regarding long-term survival and stability.

The focused inclusion of subjects presenting with a highly atrophic subantral bone condition was probably too ambitious for the hydrodynamic sinus lift approach, although the study design does not allow us to distinguish between causalities of the failed success in the tSFE test group.

Future investigations should therefore aim at larger RCTs to minimize patient-related variability. Longer follow-up periods are warranted to monitor the long-term behavior of implants that are placed in such grafted sites.

## 5. Conclusions

With these limitations in mind, the present findings indicate that the use of xHyA together with β-TCP/HA in SFE promotes rapid graft remodeling and leads to a high proportion of vital bone after 5 months of integration. This grafting protocol appears to be safe and clinically reliable for a rather conservative approach. The transcrestal access option may represent a limitation for successful grafting with this biomaterial combination, since the resorption rate in this subgroup was above average.

## Figures and Tables

**Table 1:** Inclusion and exclusion criteria of this clinical trial.

Inclusion criteria	Patients aged between 18 and 80 Patients who wished for an implant-based restoration in the posterior upper jaw ≤4 mm residual bone height
Exclusion criteria	Questionable compliance Patients with systemic, general medicine diseases (coronary heart disease, rheumatoid form circle, COPD, Diabetes Mellitus type 2 with HbA1c < 6.5%) Pregnancy/lactation Patients with cognitive/physical disturbance Patients who underwent radiotherapy affecting the head and neck area Patients who had an intravenous perfusion of bisphosphonates Diseases affecting sinus aeration Patients with a previous radical operation of the maxillary sinus, such as Caldwell-Luc Tumor alteration

**Table 2:** Demographic data of the 20 patients, subdivided into donors and non-donors.

	Donor	Non-Donor
**Age in years**	60.4 ± 10.21	57 ± 7.18
**Gender**		
Male	6	5
Female	4	5
**Smoking behavior**		
Non-smoker	6	3
Ex-smoker	4	3
Smoker	0	4
**Group**		
Test group	3	7
Control group	7	3
**RBH in mm**	2.2 ± 0.96	2.6 ± 0.8
**Region allocated to tx**		
16	3	6
26	7	4

**Table 3:** Descriptive statistics of combined and grouped mean values of subantral bone height (± SD) and mean gain as Δ (± SD), excluding dropouts.

Parameter (*n* = 18)	Combined Mean (mm ± SD)	Test Group (*n* = 8) (mm ± SD)	Control Group (*n* = 10) (mm ± SD)
Baseline RBH (3D)	2.5 ± 0.90	2.49 ± 1.03	2.48 ± 0.81
Total Bone Height Pre-Implantation (3D)	11.0 ± 5.44	5.86 ± 1.48	15.11 ± 3.47
Δ Pre-Implantation (mm gain)	8.5 ± 5.57	3.38 ± 1.32	12.63 ± 3.87

**Table 4:** Histomorphometric assessment of new bone formation (NB), connective tissue (CT), and residual bone substitute material (RG), calculated as percentage of total tissue area.

Proband-ID	Group	NB (%)	CT (%)	RG (%)
1	C	24	31.967	50.5
2	T	52.6245	36.1345	11.241
6	C	47.8145	46.955	5.2305
7	C	72.3725	27.6275	0
8	C	69.2885	21.831	8.82
10	T	57.3945	42.6055	0
11	C	63.7945	33.5385	2.892
15	T	81.1965	18.8035	0
17	C	72.838	15.145	10.517
22	C	70.3415	29.6585	0

**Table 5:** Immunohistochemistry (IHC) and enzyme histochemistry: Semi-quantitative analysis based on the percentage of positively stained area and classified as − − (0%), − (<5%), 0 (5–25%), + (25–75%), or ++ (>75%).

Proband-ID	Group	vWF	OC	OP	ALP	ED-1: Osteoclasts/ Macrophages	ED-1: MNGCs	RUNX2	COL1	TRAP
1	C	+	++	+	0	0	− −	0	+	0
2	T	0	+	+	−	− −	− −	+	+	0
6	C	+	++	+	0	−	− −	+	0	−
7	C	−	+	−	0	−	− −	−	−	−
8	C	+	0	−	0	−	− −	0	+	−
10	T	0	+	0	0	−	− −	−	0	−
11	C	−	0	−	0	− −	− −	0	−	−
15	T	+	+	−	0	−	− −	−	+	−
17	C	0	+	−	0	−	− −	−	0	−
22	C	0	+	0	+	−	− −	+	+	−

Abbreviations: vWF, 1. macrophages/osteoclasts; MNGCs, multinucleated giant cells; RUNX2, runt-related transcription factor 2; COL1, collagen type I; TRAP, tartrate-resistant acid phosphatase.

## Data Availability

All data are stored at the institution which was involved in this clinical trial and can be requested.
